# Interpreting cross-sectional comparisons of menopausal status and brain outcomes: Letter re: Emotional and cognitive effects of menopause and hormone replacement therapy

**DOI:** 10.1017/S0033291726104073

**Published:** 2026-04-07

**Authors:** Catriona Keye

**Affiliations:** General Practice, Scholarstown Family Practice, Dublin, Ireland

Dear Editor-in-Chief,

I read with interest the recent report by Zühlsdorff et al. examining associations between menopausal status, gray matter volume, sleep disturbance, and mental health outcomes in a large population-based sample (Zühlsdorff et al., 2026). The scale of the dataset and integration of neuroimaging and behavioral measures represent an important contribution. However, I wish to highlight a key limitation in interpretation that warrants clarification.

The comparison of pre- and post-menopausal participants in this study is cross-sectional and involves different individuals rather than repeated assessments of the same women. As such, observed differences reflect between-person variation rather than within-person change across the menopausal transition. Consequently, these findings cannot establish that menopause itself caused the reported differences in brain structure, sleep, or mental health.

This distinction is particularly important in midlife research. Menopause occurs against a backdrop of accumulated biological, psychosocial, and health-related exposures that vary substantially across individuals. Factors such as cardiometabolic health, psychiatric history, sleep disorders, medication use, stress burden, and socioeconomic conditions are associated with both menopausal timing and brain-related outcomes (Gold et al., 2013). Even with statistical adjustment, residual confounding and baseline heterogeneity remain unavoidable in cross-sectional designs, limiting causal inference (Hernán & Robins, 2020; Salthouse, 2011).

Longitudinal research across the menopausal transition provides a contrasting perspective. Syntheses led by Maki and colleagues indicate that menopause-related cognitive changes are generally modest, stage-dependent, and heterogeneous, with evidence of stabilization or improvement following the transition in many women (Maki, 2015; Maki & Thurston, 2020). This literature emphasizes the role of menopausal symptoms – particularly vasomotor symptoms, sleep disturbance, and mood changes – as potential mediators of cognitive outcomes, rather than menopause exerting a uniform direct neurobiological effect (Maki, 2015).

I, therefore, suggest that conclusions drawn from cross-sectional comparisons be framed explicitly as associations with menopausal status, rather than as evidence of menopause-related neurobiological change. Longitudinal, within-person neuroimaging studies spanning defined menopause stages are required to determine whether and how menopause contributes to structural brain change. Careful alignment between study design and interpretation will strengthen the evidence base and support accurate clinical and public health communication.

Yours Sincerely

Catriona Keye

BMS Menopause Specialist

## Declarations

The author has not received any funding for this letter to the editor. The author studies at the University College Cork as a PhD candidate; however, this work was not a part of the studies. The author declares no conflicts of interest.
